# Cross-modal motion aftereffects transfer between vision and touch in early deaf adults

**DOI:** 10.1038/s41598-021-83960-0

**Published:** 2021-02-23

**Authors:** Kunchen Xiao, Yi Gao, Syed Asif Imran, Shahida Chowdhury, Sesh Commuri, Fang Jiang

**Affiliations:** 1 Institute of Brain and Psychological Sciences, Sichuan Normal University, Chengdu, 610066 Sichuan Province China; 2 Department of Psychology, University of Nevada, Reno, NV 89557-0296 USA; 3 Department of Electrical and Biomedical Engineering, University of Nevada, Reno, NV 89557-0260 USA

**Keywords:** Human behaviour, Auditory system, Cognitive neuroscience, Diseases of the nervous system, Sensorimotor processing, Sensory processing, Somatosensory system, Visual system, Neuroscience, Psychology

## Abstract

Previous research on early deafness has primarily focused on the behavioral and neural changes in the intact visual and tactile modalities. However, how early deafness changes the interplay of these two modalities is not well understood. In the current study, we investigated the effect of auditory deprivation on visuo-tactile interaction by measuring the cross-modal motion aftereffect. Consistent with previous findings, motion aftereffect transferred between vision and touch in a bidirectional manner in hearing participants. However, for deaf participants, the cross-modal transfer occurred only in the tactile-to-visual direction but not in the visual-to-tactile direction. This unidirectional cross-modal motion aftereffect found in the deaf participants could not be explained by unisensory motion aftereffect or discrimination threshold. The results suggest a reduced visual influence on tactile motion perception in early deaf individuals.

## Introduction

Research on early deafness has primarily focused on the behavioral and neural changes in the intact visual and tactile modalities. The visual abilities of deaf individuals have been extensively studied to understand the plasticity of multimodal perception following auditory deprivation. Early deaf individuals show enhanced detection and discrimination of directional visual motion^1,2^, especially in the periphery vision^3,4^. The enhanced visual abilities as a result of auditory deprivation are often accompanied by cross-modal recruitment of auditory cortex^5,6^. Increased neural responses to visual motion in the deaf have been reported in multisensory areas, such as the superior temporal sulcus (STS)^7,8^ and superior temporal gyrus (STG)^9,10^, in line with animal research showing increased neurons in multimodal regions responsive to the intact modality after hearing loss^11^.

On the other hand, whether tactile processing is enhanced or impaired after hearing loss is inconclusive. For example, compared to hearing controls, deaf individuals showed enhanced accuracy in tactile frequency detection^12^. However, other research reported that deaf individuals showed no enhancement of overall tactile processing and even impaired performance in tactile temporal discrimination tasks^13–17^. The change in tactile performance due to early deafness may depend on the nature of the task: if the tasks involve the temporal dimension, a dimension that is typically associated with hearing, performance impairments could be observed in deaf individuals.

Cross-modal reorganization within primary auditory and multimodal regions is likely to influence not only the processing ability within the visual and tactile modalities but also the interaction between them. Neuroimaging findings suggest that multisensory interactions occur in the auditory cortex^18^ and multisensory areas^19,20^ in hearing individuals. In deaf individuals, the auditory cortex was activated in tactile^21^ and visual processing^5^ and showed greater neural responses to somatosensory stimulation^22,23^. Since neural reorganizations took place in auditory cortex and multisensory areas as a result of hearing loss^7,9,10^, it is reasonable to hypothesize that auditory deprivation may change the way vision and touch interact within these areas. However, the visuo-tactile interaction in deaf individuals has not been well studied, and results from limited research seem contradictory. Deaf individuals showed an increased visual influence on touch for spatial processing in visuo-tactile conflict when judging the spatial location of stimuli^24^. In contrast, deaf individuals were more susceptible to a double-flash visual illusion induced by two touches to the face presented in a narrow temporal window^23^, suggesting a dominance of touch over vision when the task is reliant on temporal processing.

This discrepancy may be attributed to the distinction between spatial and temporal processing during visuo-tactile interactions in deaf individuals. Vision may dominate spatial tasks over touch and hearing, since vision typically provides the most reliable information for spatial perception^25^. Contrarily, touch and hearing may dominate temporal tasks over vision^14,26,27^. When auditory processing is deprived, temporal processing may become more dependent on the tactile modality. This interpretation is consistent with the modality appropriateness hypothesis^28^, whereby sensory information is weighted according to the relative relevancy of the information conveyed by each modality^29^; the sensory modality that weights more in a particular context will dominate behaviors^30^. For example, tactile motion perception requires discriminating the temporal order of skin deformation and thus may weight more on temporal than spatial processing^19^.

To further test this hypothesis, in the present study, we examined the effect of hearing loss on the visuo-tactile interaction using a cross-modal motion adaptation paradigm. It has been demonstrated that motion aftereffects transfer bi-directionally between vision and touch in normal-hearing individuals: adapting to visual motion in one direction causes a subsequent tactile stimulus to be perceived as moving in the opposite direction (visual-to-tactile aftereffect) and vice versa (tactile-to-visual aftereffect)^31^. Visual and tactile motion perceptions involve similar mechanisms of processing spatiotemporal patterns of activation across populations of sensory receptors: the motion direction is first determined by local motion detectors and then over time integrated to develop a global motion perception^20,32,33^. The cross-modal motion aftereffect involves neural substrates shared by visual and tactile motion processing^19,20^, including STS^34^, MST^35^, posterior parietal cortex^36^, and human motion complex hMT + /V5^37–40^.

In Experiment 1, we adopted the procedure from the study by Konkle et al.^31^. By comparing the cross-modal motion adaptation between hearing and deaf individuals, the present study seeks to understand how early deafness affects the transfer of motion aftereffects between vision and touch. If discriminating the direction of tactile motion (tactile motion stimuli are sequentially presented with varying inter-stimulus onset between vibrating rows) relies on temporal processing, and temporal processing becomes more reliant on the tactile channel after the auditory channel is deprived, tactile motion perception would weight more on touch and less on vision in deaf individuals. Thus, deaf individuals’ tactile motion perception would be less affected by visual adaptation, reducing the visual-to-tactile interference. Therefore, we would expect reduced or even absent visual-to-tactile motion aftereffects in deaf individuals, while tactile-to-visual motion aftereffects would be similar to those reported in hearing controls^31^. In Experiment 2, we assessed whether the transfer pattern we found in Experiment 1 could be explained by the difference in unisensory motion aftereffects or discrimination thresholds between the deaf and hearing groups.

## Experiment 1

### Participants

Ten participants with severe to profound early deafness (age 32–67; *M* = 44.4, *SD* = 12.38, 2 males) and ten hearing participants (age 31–66; *M* = 44.8, *SD* = 12.88, 2 males) were recruited from Reno and surrounding areas. All deaf participants became deaf before the age of 2 and were fluent in American Sign Language (See Table 1 for characteristics of deaf participants). None of the deaf use hearing-aids. Deaf and hearing participants were matched on gender and age. All participants were right-handed and had a normal or corrected-to-normal vision.

**Table 1:** The characteristics of deaf participants.

Subject	Gender	Age	Degree of deafness (dB)	Age acquiring sign language	Age becoming deaf	Cause of deafness
1	Female	32	96	1 year	Born	Unknown
2	Female	34	80	1 year	Born	Hereditary
3	Female	67	90	5 years	Born	Unknown
4	Female	60	100	5 years	Born	Unknown
5	Female	40	90	2 years	Born	Genetic
6	Female	53	85	8 years	Born	Unknown
7	Male	35	91	2 years	Born	Cytomegalovirus
8	Male	32	85	15 months	15 months	Fever
9	Female	49	90	11 years	Born	Mom measles during pregnancy
10	Female	42	95	12 years	12 months	Unknown

We calculated the effect size based on the smallest visuo-tactile aftereffect reported in the study by Konkle et al.^31^, and the estimated Cohen’s *d* was 1.74. With this conservative estimation of effect size, our prospective power analysis showed that a sample size of ten could detect a visuo-tactile aftereffect of 1.74 at an alpha level of 0.05 with a power of 0.82.

Participants provided signed informed consent before any experimentation. All experiment protocols were reviewed and approved by the Institutional Review Board at the University of Nevada, Reno. The study was conducted in accordance with the Declaration of Helsinki.

### Apparatus

**Figure 1:**
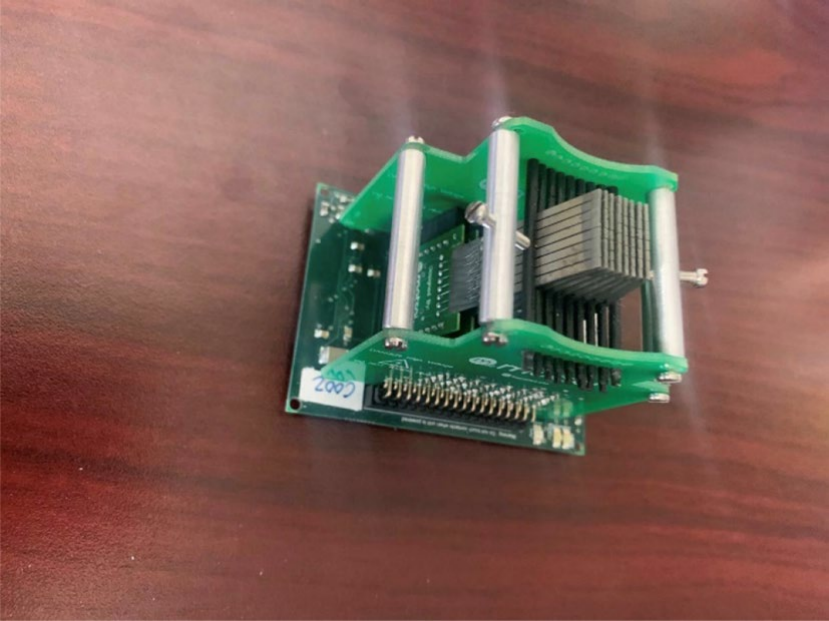
A photo of the Latero device without the external plastic cover. On the top of the device is an array of 8 × 8 pins, where participants place their fingertips to feel the tactile vibration.

Tactile stimuli were generated by the Latero (http://tactilelabs.com/), a state-of-the-art tactile display that deforms the finger pad skin with an array of laterally vibrating pins actuated by miniature piezoelectric bending motor^41^. It has an array of 8 × 8 pins on the surface, with 1.2 × 1.6 mm center-to-center pin spacing and 1.2 cm^2^ total active area (Fig. 1). Each pin can independently vibrate at frequencies up to 100 Hz. By customizing the frequency and duration of vibration for each pin, a tactile motion stimulus can be delivered to participants’ fingertips. During the experiments, the Latero was located 15 cm behind and 15 cm below the center point of a 60 Hz Macintosh LED monitor. The screen center and the Latero array were placed in line so that the fingertip was positioned in the foveal visual field to approximate the best spatial correspondence between the visual fixation and the fingertip.

### Method

The experiment contained two within-subject conditions: a visual-to-tactile (VT) condition and a tactile-to-visual (TV) condition. The order of the two conditions was counterbalanced between participants, and there was a twenty-minute break between the two conditions. Both conditions consisted of four sessions: a practice session, a baseline session, an adapt-to-upward-motion session, and an adapt-to-downward-motion session.

*Visual-to-tactile (VT) Condition*. There were two blocks in the practice session. In Block 1, eight successive rows or columns of tactile vibration were delivered (each row or column vibrated for 30 ms at 33.3 Hz) to produce a tactile “sweep” motion, randomly in one of the four directions: upward, downward, leftward, or rightward. Participants reported the motion direction by pressing “U” for upward, “D” for downward, “L” for leftward, and “R” for rightward on the keyboard, and were given feedback for incorrect responses. There were two sets of 20 sweeps.

In Block 2, 20 sweeps either upward or downward were given. Participants pressed “U” for upward motion and “D” for downward motion and received feedback for each response. Each sweep consisted of four tactile rows (row 1, 3, 5, and 7) and the temporal gap between two consecutive rows varied from − 40 to 40 ms, with a positive number for upward motion and a negative number for downward motion. Participants were then informed that although it was relatively easy to tell the directions of these tactile sweeps, the Latero could produce ambiguous sweeps that were difficult to judge, which would be presented for the rest of the experiment.

In the baseline session, tactile motion direction discrimination was measured without adaptation. Participants were asked to judge whether a tactile sweep was moving upward or downward. Each participant completed three blocks and each block had one staircase. The tactile sweep consisted of four vibrating tactile rows (Row 1, 3, 5, and 7) in sequence and each row vibrated for 100 ms at 33.3 Hz; The temporal gap between the onsets of two consecutive rows (inter-stimulus onset, ISO) was manipulated by a 1-down-1-up staircase procedure to determine the motion direction: a positive ISO represented an upward sweep while a negative ISO represented a downward sweep, and 0 ms ISO indicated that the four rows started vibrating simultaneously (no motion direction). The staircases started from a 1 ms ISO with a 2 ms step and stopped when 20 reversals or 40 trials were reached. That means the ISO would increase by 2 ms following an incorrect response and decrease by 2 ms following a correct response. And the reversal was defined as a turning point, which was the trial when the ISO changed from being increased to being decreased, or vice versa. Participants were instructed to press the “U” for upward sweeps and “D” for downward sweeps for each trial.

**Figure 2:**
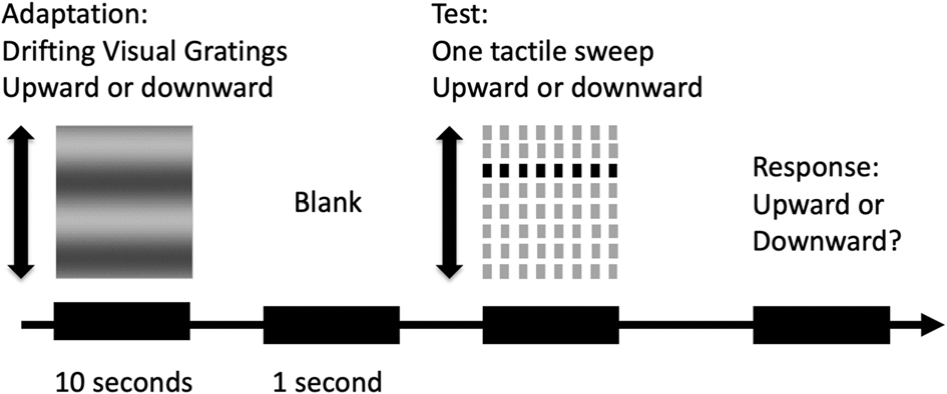
The experimental procedure in the visual-to-tactile condition. In each trial, a 10-s visual grating drifting either upward or downward was presented, followed by a period of 1 s blank and then a rapid target tactile sweep delivered to participants’ index fingertip of the right hand. The duration of the tactile sweep was determined by the staircase procedure. Participants were asked to judge the motion direction of the tactile sweep and press “U” for upward motions and “D” for downward motions.

In the adaptation sessions, participants were presented with a visual grating with fixed parameters (10% Michelson contrast, a spatial frequency of 1.05 cycles per degree, a temporal frequency of 2 Hz, subtending 1.9 × 2.3 degrees in the central visual field with a black fixation dot in the center) drifting either upward or downward for 10 s, followed by a blank screen for 1 s, and then a target tactile sweep. The target tactile sweep had the same parameters as those in the baseline session, and its ISO (ms) was manipulated by the same 1-down-1-up staircase procedure. Participants judged the tactile motion direction by either pressing the “U” button for upward or “D” button for downward motions (Fig. 2). No feedback was given. There were two sessions with a ten-minute break in between: one session adapting to upward drifting gratings and the other adapting to downward drifting gratings. The order of the two sessions was counterbalanced between participants.

*Tactile-to-visual (TV) Condition*. For practice, participants first completed the same Block 1 as in the visual-to-tactile condition. Participants then received 20 tactile sweeps (one sweep per second) randomly in one of the four directions: upward, downward, leftward, and rightward. The parameters of the sweeps were the same as in Block 1. At the same time, a “matched” visual grating, which was the same in parameters as in the visual-to-tactile condition, was presented with the same onset and ending time and in the same direction as the tactile sweep. Participants were asked to observe the visual grating, feel the tactile sweeps, and imagine that their index finger was aligned with the fixation point.

In the baseline session, participants completed three staircases of drifting gratings by judging whether the grating was moving upward or downward. The grating was the same in parameters as in the visual-to-tactile condition except that the contrast was reduced to 1%. The grating consisted of 5 successive frames, and each frame lasted for 200 ms (1 s in total). The phase jump between two successive frames was manipulated by a 1-down-1-up staircase procedure. A perfect counter-phase of the original grating was defined as 180°; a positive phase jump represented an upward drifting while a negative phase jump represented a downward drifting. The three staircases respectively started from a phase jump of 40°, − 40°, or 0°, with a step size of 4°. The staircase stopped when 20 reversals or 40 trials were reached. Participants were asked to judge the visual motion direction by pressing “U” for “upward” and “D” for “downward.”

**Figure 3:**
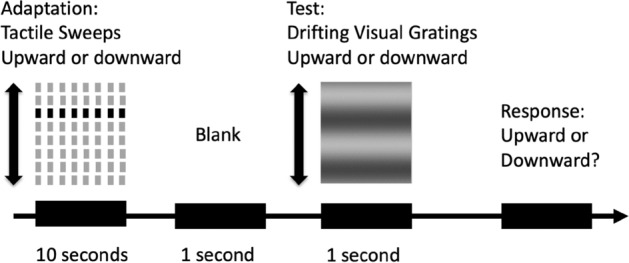
The experimental procedure in the tactile-to-visual condition. In each trial, 20 successive tactile sweeps, all going either upward or downward, were delivered to participants’ index fingertip of the right hand for 10 s, followed by 1 s blank and then a target visual grating drifting for 1 s. Participants were asked to judge the motion direction of the drifting grating and press “U” for upward motions and “D” for downward motions.

In the adaptation sessions, participants adapted to tactile sweeps in one direction and then judged the motion direction of a drifting grating. In each trial, participants first received 20 successive tactile sweeps with fixed parameters for a total of 10 s: a single 240 ms sweep consisted of all 8 rows and each row vibrated for 30 ms at 33.3 Hz (i.e., ISO = 30 ms), followed by a 260 ms pause. All 20 tactile sweeps were going in the same direction: either upward or downward. Right after the 20 sweeps was a blank period for 1 s. Then a target drifting grating was presented for 1 s in the center of the screen, which was the same in parameters as in the baseline session. Participants judged the motion direction of the target grating by pressing “U” for “upward” and “D” for “downward” motions (Fig. 3). The phase jump of the visual grating always started from 2° and was manipulated by the same 1-down-1-up staircase procedure as in the baseline session. There were two sessions with a ten-minute break in between: one session adapting to upward tactile sweeps and the other adapting to downward sweeps. The order of the two sessions was counterbalanced between participants.

### Results

Following the Konkle et al. study^31^, the point of subjective equality (PSE) for each participant was calculated by discarding the first two reversals and averaging the ISO or phase jump of the remaining reversals. The divergence of staircases after adaptations was estimated by the difference of PSEs between upward-motion adaptation and downward-motion adaptation. This measure reflects the effect of adaptation because the two staircases starting from the same seed would not diverge if there were no aftereffects at all^31^. Therefore, the magnitude of the aftereffect was quantified as the difference between the PSE adapt-to-upward-motion and the PSE adapt-to-downward-motion. In addition to the divergence of the two staircases, we compared PSE measured in each staircase to those measured in the baseline session to examine the aftereffect caused by adaptation to each direction. To examine the impact of hearing loss on motion adaptation, a two-way ANOVA on PSE was performed separately for the visual-to-tactile (VT) and tactile-to-visual (TV) conditions with adapting direction (upward vs. downward) as a within-subject factor and group (deaf vs. hearing) as a between-subject factor. For multiple testing correction, we applied Bonferroni adjustment. Specifically, following each ANOVA analysis, there were four Post-Hoc t-tests, which were treated as one family of t-tests. Thus, the adjusted *p-*value for significance was 0.0125 (0.05/4) for t-tests in the same family.

**Figure 4:**
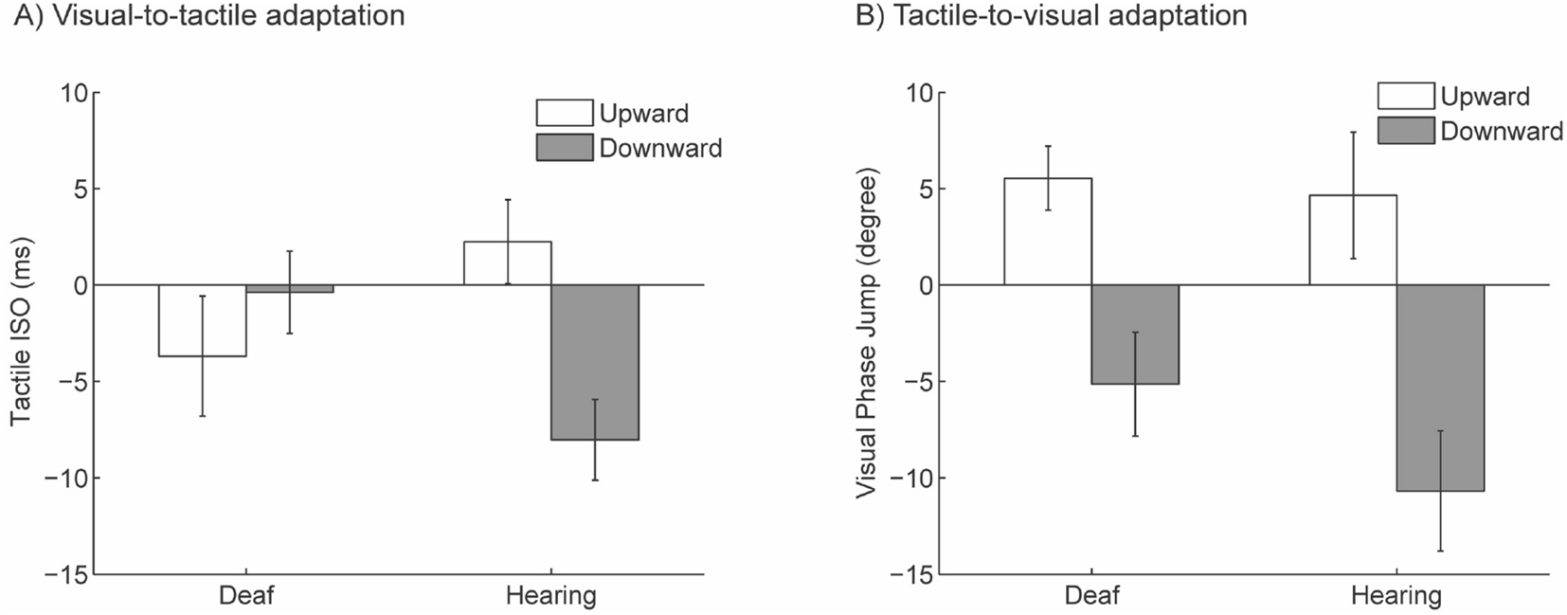
Cross-modal aftereffects measured by PSE in Experiment 1. (Panel **A**) shows the average of tactile PSEs after adaptation to visual motions, while (Panel **B**) shows the average of visual PSEs after adaptation to tactile motions. PSE after the upward and downward adaptation are shown respectively in white and grey. Error bars represent one standard error.

*Visual-to-tactile adaptation*. The ANOVA showed that there was no main effect of adapting direction (*F*(1, 18) = 2.29, *P* > 0.14, partial *η*^2^ = 0.11) or group (*F*(1, 18) = 0.11, *P* > 0.73, partial *η*^2^ = 0.006). A significant interaction was found between adapting direction and group (*F* (1, 18) = 8.71, *P* = 0.009, partial *η*^2^ = 0.33). Post-Hoc t-tests indicate that the aftereffect was significant in the hearing group: the PSE of upward adaptation was significantly higher than that of the downward adaptation in hearing individuals (*t* (9) = 4.84*, P* < 0.001, Cohen’s *d* = 1.61). However, for the deaf group, the PSE of upward adaptation was not significantly different from the PSE of downward adaptation (*t* (9) = 0.81*, P* > 0.43, Cohen’s *d* = 0.27), indicating no significant visual-to tactile motion aftereffect (Fig. 4).

**Figure 5:**
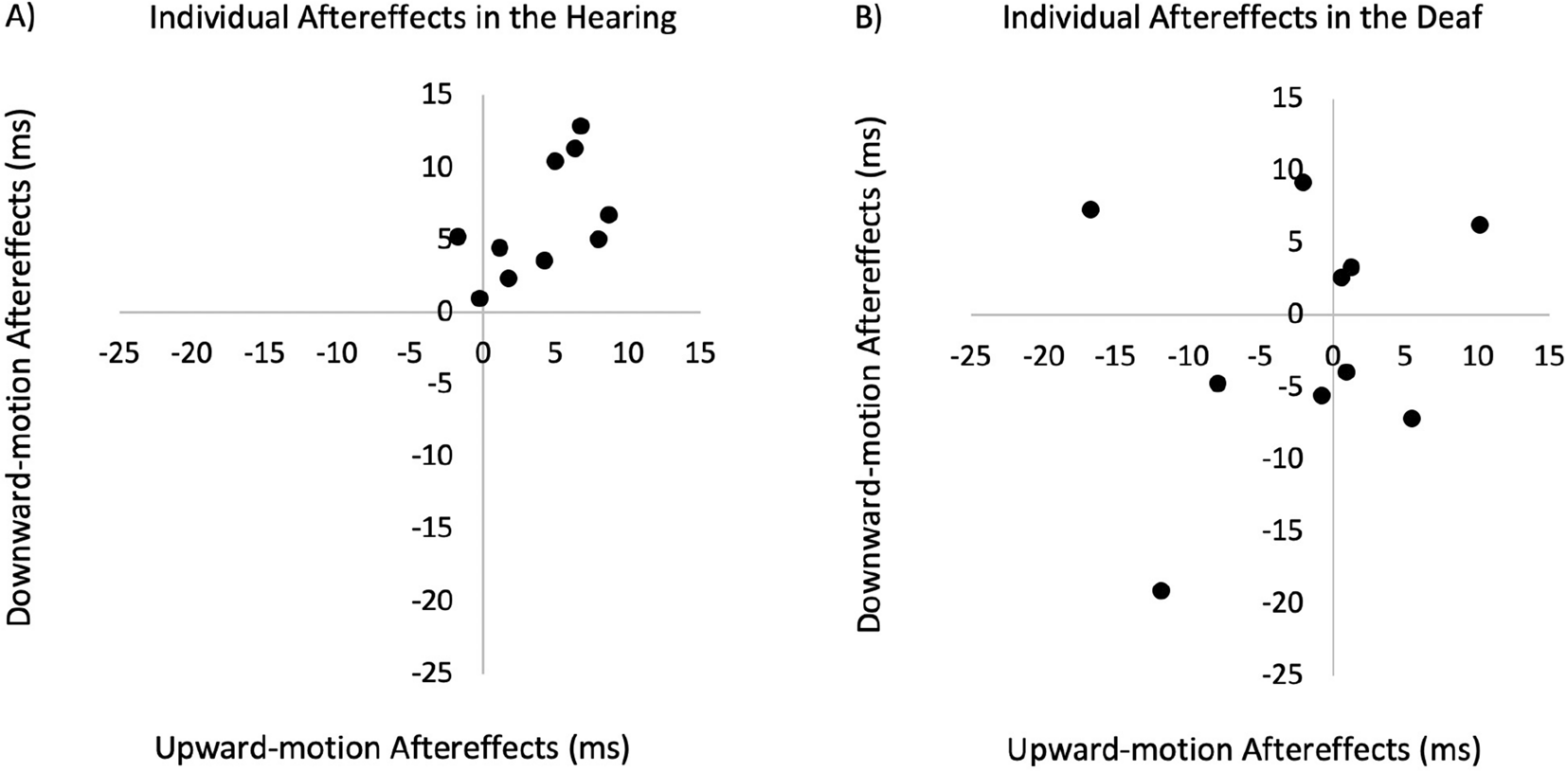
Scatter plots showing individual visual-to-tactile aftereffects in hearing (Panel **A**) and deaf (Panel **B**) group. The x-axis represents upward-motion aftereffect (upward-adaptation PSE-baseline PSE), and the y-axis plots the downward-motion aftereffect (baseline PSE-downward-adaptation PSE). The unit is ms of tactile ISO. Note that both upward-motion and downward-motion aftereffects plotted here are expected to be positive values.

**Figure 6:**
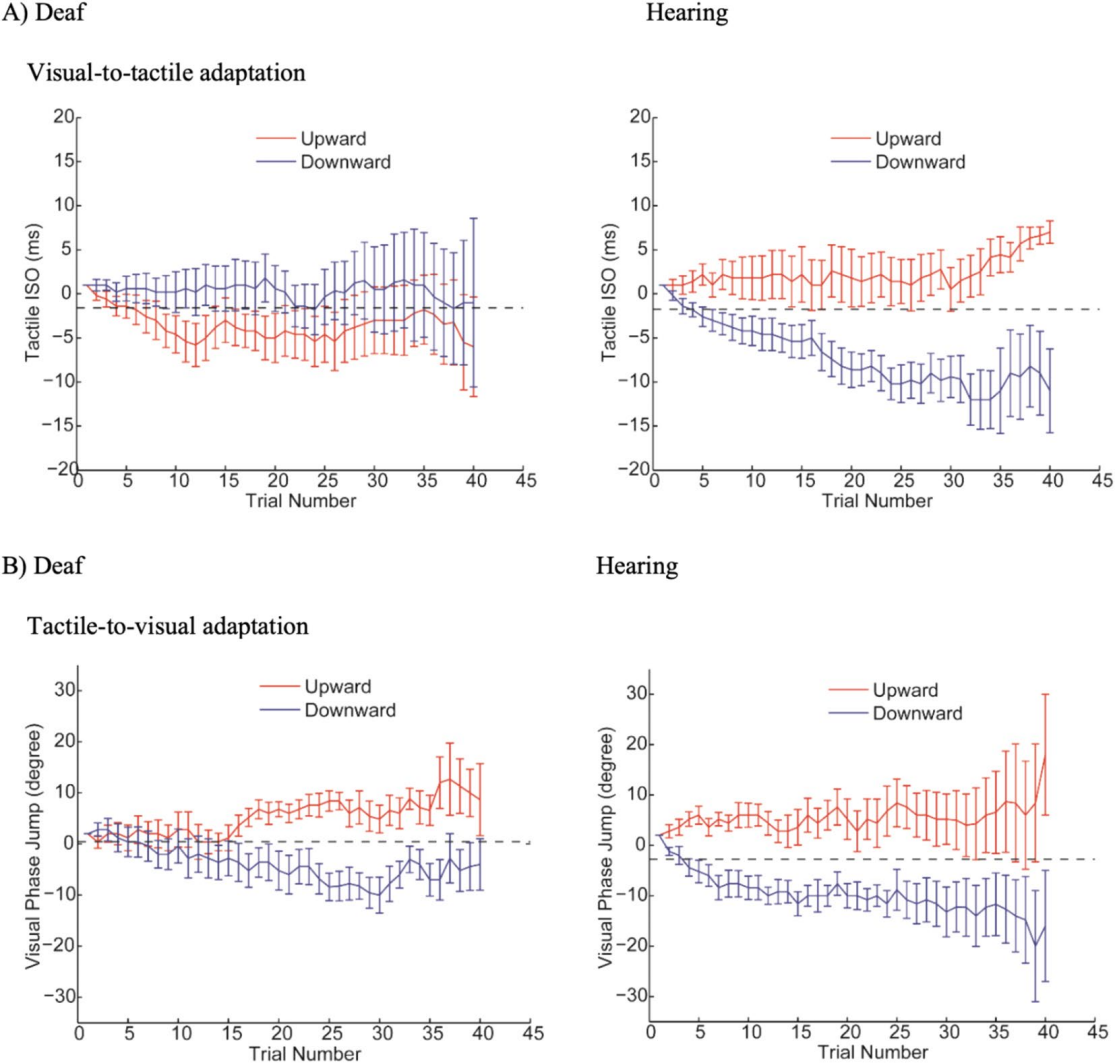
Staircase progress in Experiment 1. The staircase progress is shown separately for the deaf (left column) and hearing participants (right column) in the visual-to-tactile (panel **A**) and tactile-to-visual (panel **B**) adaptation conditions. Staircases in the upward and downward adaptation conditions are shown respectively in red and blue. The black dashed lines were the average baseline PSE without adaptation. Error bars represent one standard error.

In the hearing group, PSE after adaptation was significantly different from the baseline PSE (upward adaptation: *t*(1, 9) = 3.52, *P* < 0.007, Cohen’s *d* = 1.17; downward adaptation: *t*(1, 9) = 4.97, *P* < 0.001, Cohen’s *d* = 1.65). In contrast, in the deaf group, neither PSE after adapting to upward or downward was significantly different from baseline PSE (upward adaptation: *t*(1, 9) = 0.83, *P* > 0.42, Cohen’s *d* = 0.27; downward adaptation: *t*(1, 9) = 0.44, *P* > 0.66, Cohen’s *d* = 0.14) (Fig. 4A). Individual data of aftereffects are shown on the scatter plots below for hearing (Fig. 5A) and deaf (Fig. 5B) participants. As demonstrated by the staircase progress in Fig. 6A, a significant divergence between upward and downward adaptation was observed in the hearing group (Sign test, *P* = 0.002) but not in the deaf group (Sign test, *P* > 0.75).

In addition, there was no significant difference of baseline PSE between the hearing (*M* = − 1.73, *SD* = 5.58) and deaf (*M* = − 1.58, *SD* = 4.59); *t*(1, 9) = 0.06, *P* > 0.95. To check the order effect of adaptation tasks, both hearing and deaf groups were further divided into two subgroups according to the order of tasks performed: one group first performed the visual-to-tactile task while the other group first performed the tactile-to-visual task; the aftereffects of visual-to-tactile adaptation were compared between the two subgroups, and no order effect was found for the hearing or the deaf group (*P* > 0.25). These results suggest that the presence of visual-to-tactile motion aftereffects in hearing but not in deaf participants was not due to baseline performance or testing order. In addition, the Pearson’s Correlation Coefficient between the severity of deafness and the aftereffect was not significant (*r*(9) = − 0.52, *P* = 0.12), suggesting the degree of hearing loss has a nonsignificant influence on the magnitude of visual-to-tactile motion aftereffect.

*Tactile-to-visual adaptation*. The ANOVA showed a significant main effect of direction (*F*(1, 18) = 23.44, *P* < 0.001, partial *η*^2^ = 0.57). Neither the main effect of group (*F*(1, 18) = 1.29, *P* > 0.27, partial *η*^2^ = 0.07) nor the interaction effect between adapting direction and group (*F*(1, 18) = 0.75, *P* > 0.39, partial *η*^2^ = 0.04) was significant. Post-Hoc t-test showed significantly higher PSE after upward adaptation than downward adaptation for both hearing (*t*(9) = 3.45, *P* = 0.007, Cohen’s *d* = 1.52) and deaf individuals *t*(9) = 3.53, *P* = 0.006, Cohen’s *d* = 1.51), indicating significant aftereffects in both groups.

**Figure 7:**
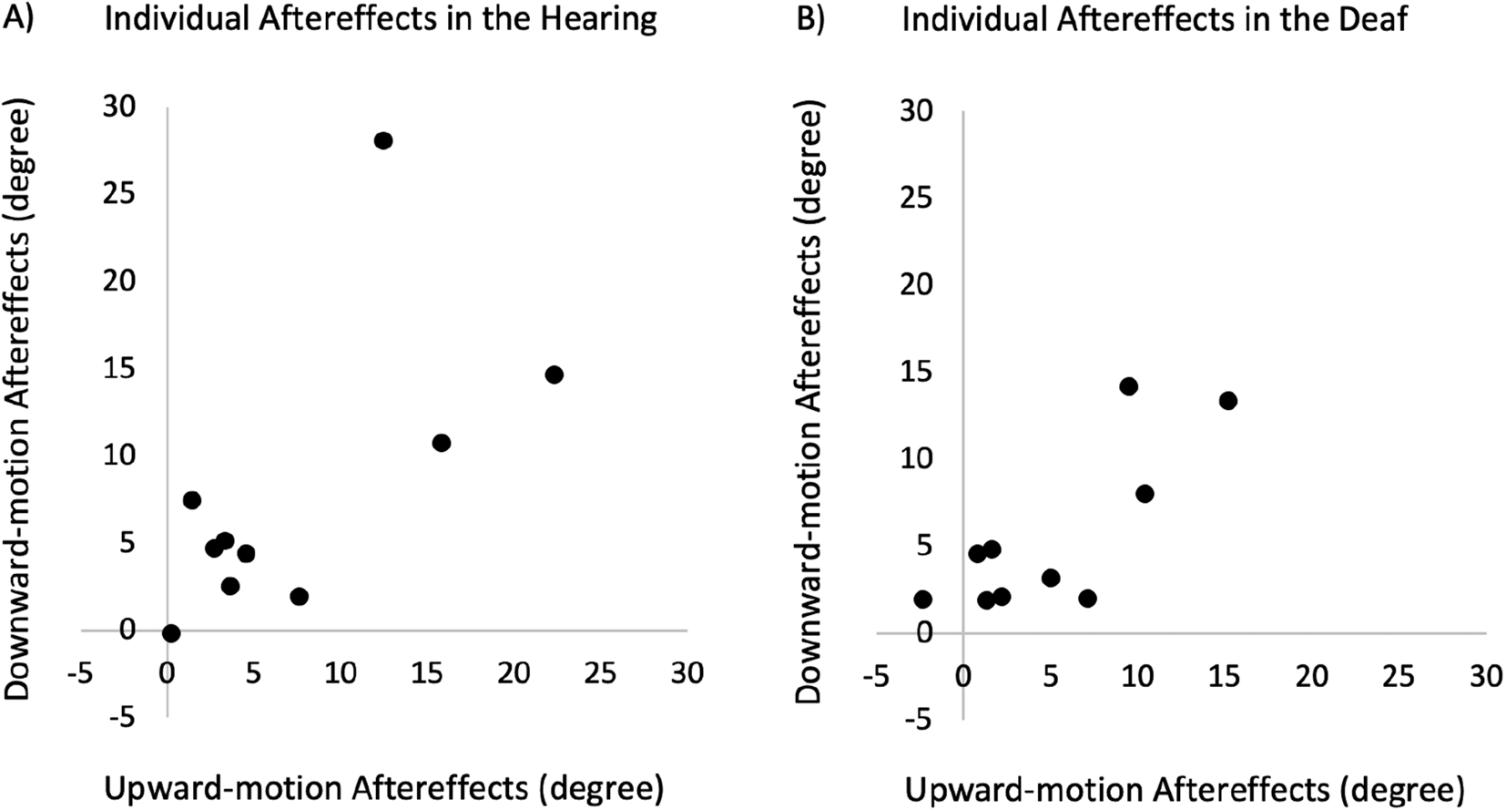
Scatter plots showing individual tactile-to-visual aftereffects in the hearing (Panel **A**) and deaf (Panel **B**) group. The x-axis represents upward-motion aftereffects (upward-adaptation PSE-baseline PSE), and the y-axis represents downward-motion aftereffects (baseline PSE-downward-adaptation PSE). The unit is degree of visual phase jump. Note that both upward-motion and downward-motion aftereffects plotted here are expected to be positive values.

As shown in Fig. 6B, a significant divergence between PSE after upward adaptation and downward adaptation was present in both hearing (Sign test, *P* = 0.002) and deaf group (Sign test, *P* < 0.03). In the hearing group, PSE after adaptation was significantly different from the baseline (upward adaptation: *t*(1, 9) = 3.26, *P* < 0.02, Cohen’s *d* = 1.08; downward adaptation: *t*(1, 9) = 3.02, *P* < 0.02, Cohen’s *d* = 1.01). This was also true for the deaf group (upward adaptation: *t*(1, 9) = 2.97, *P* < 0.02, Cohen’s *d* = 0.99; downward adaptation: *t*(1, 9) = 3.76, *P* < 0.005, Cohen’s *d* = 1.25) (Fig. 4B). Individual data of aftereffects are shown on the scatter plots below for the hearing (Fig. 7A) and deaf (Fig. 7B) participants. See Supplementary Data online for individual PSEs of baselines and adaptations in all conditions.

In addition, there was no significant difference of baseline PSE between the hearing (*M* = − 2.74, *SD* = 5.17) and deaf (*M* = 0.46, *SD* = 5.50); *t*(1, 9) = 1.29, *P* > 0.22. Similar to the visual-to-tactile adaptation condition, no order effect of adaptation tasks was found for the hearing or the deaf group (*p*s > 0.40), and the correlation between the severity of deafness and the aftereffect was not significant (*r*(9) = − 0.49, *P* = 0.15).

## Interim discussion

The cross-modal adaptation effect reported by Konkle et al.^31^ was replicated in the hearing group. On the other hand, deaf participants showed a unidirectional aftereffect: the motion aftereffect transferred from touch to vision but not from vision to touch. The results corroborate our hypotheses that tactile motion perception, which relies on temporal cues, became less susceptible to visual influence after hearing loss. However, there could be two alternative explanations. First, congenitally deaf participants may possess enhanced sensitivity to tactile motions, which helps resisting aftereffects caused by visual adaptation. Second, differences in cross-modal aftereffects may be partially driven by changes in aftereffects within each modality. Thus, we conducted Experiment 2 to investigate these two alternative explanations by comparing discrimination thresholds of motion-direction and unisensory aftereffects between the deaf and hearing participants.

## Experiment 2

### Method

Experiment 2 examined whether the deaf and hearing participants differed in unisensory adaptation or discrimination thresholds of motion-direction. The same participants as in Experiment 1 performed two unisensory adaptation tasks: a tactile-to-tactile (TT) task and a visual-to-visual (VV) task. Each task consisted of four sessions: a practice session, a baseline session, an adapt-to-upward-motion session, and an adapt-to-downward-motion session. Participants’ discrimination thresholds of motion direction were measured with a 2-down-1-up staircase.

*Tactile-to-tactile (TT) Condition*. The procedure and testing stimulus were the same as in the visual-to-tactile adaptation in Experiment 1, and the adapting stimulus was the tactile adaptor used in the tactile-to-visual adaptation.

*Visual-to-visual (VV) Condition*. The procedure and testing stimulus were the same as in the tactile-to-visual adaptation in Experiment 1, and the adapting stimulus was the visual adaptor used in the visual-to-tactile adaptation.

*Tactile and visual discrimination thresholds of motion-direction*. The thresholds were measured separately for tactile and visual conditions. In each condition, there were three blocks. In each block, a 2-down-1-up staircase containing 80 trails was employed to measure the discrimination threshold of motion direction. The tactile motion staircase started either from a 20 ms or a -20 ms ISO with a step size of 1 ms. The visual motion staircase started either from a 40° or − 40° phase jump with a step size of 2°. The motion direction (upward/downward) was randomly determined for each trial. Participants were asked to judge the direction of each motion and pressed the “U” for “upward motion” and the “D” for “downward motion.” The average of the last 6 reversals of the three blocks was taken as the discrimination threshold of 70.7% correct.

### Results

A two-way ANOVA on PSE was performed separately for the tactile-to-tactile (TT) and visual-to-visual (VV) conditions with adapting direction (upward vs. downward) as a within-subject factor and group (deaf vs. hearing) as a between-subject factor.

**Figure 8:**
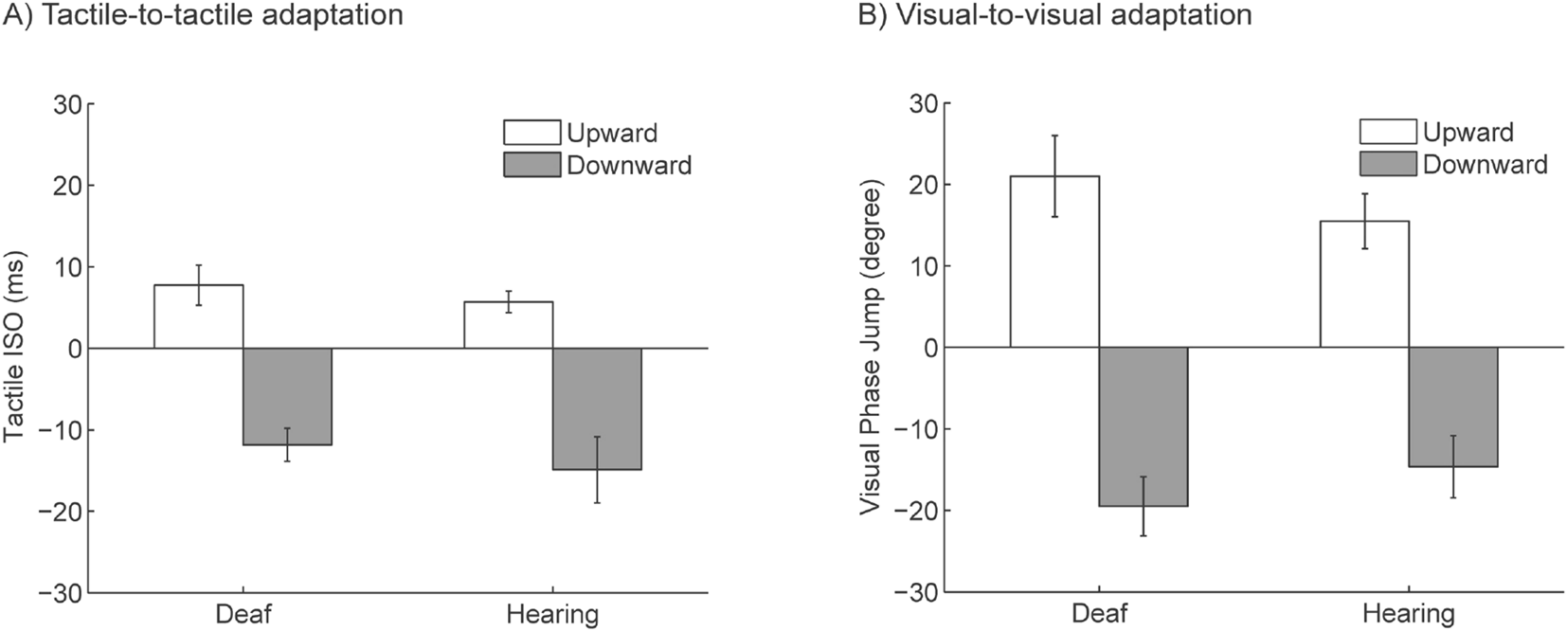
Unisensory aftereffects measured by PSE in Experiment 2. (Panel **A**) shows the average of tactile PSEs after adaptation to tactile motions, while (Panel **B**) shows the average of visual PSEs after adaptation to visual motions. PSE after the upward and downward adaptation are shown respectively in white and grey. Error bars represent one standard error.

**Figure 9:**
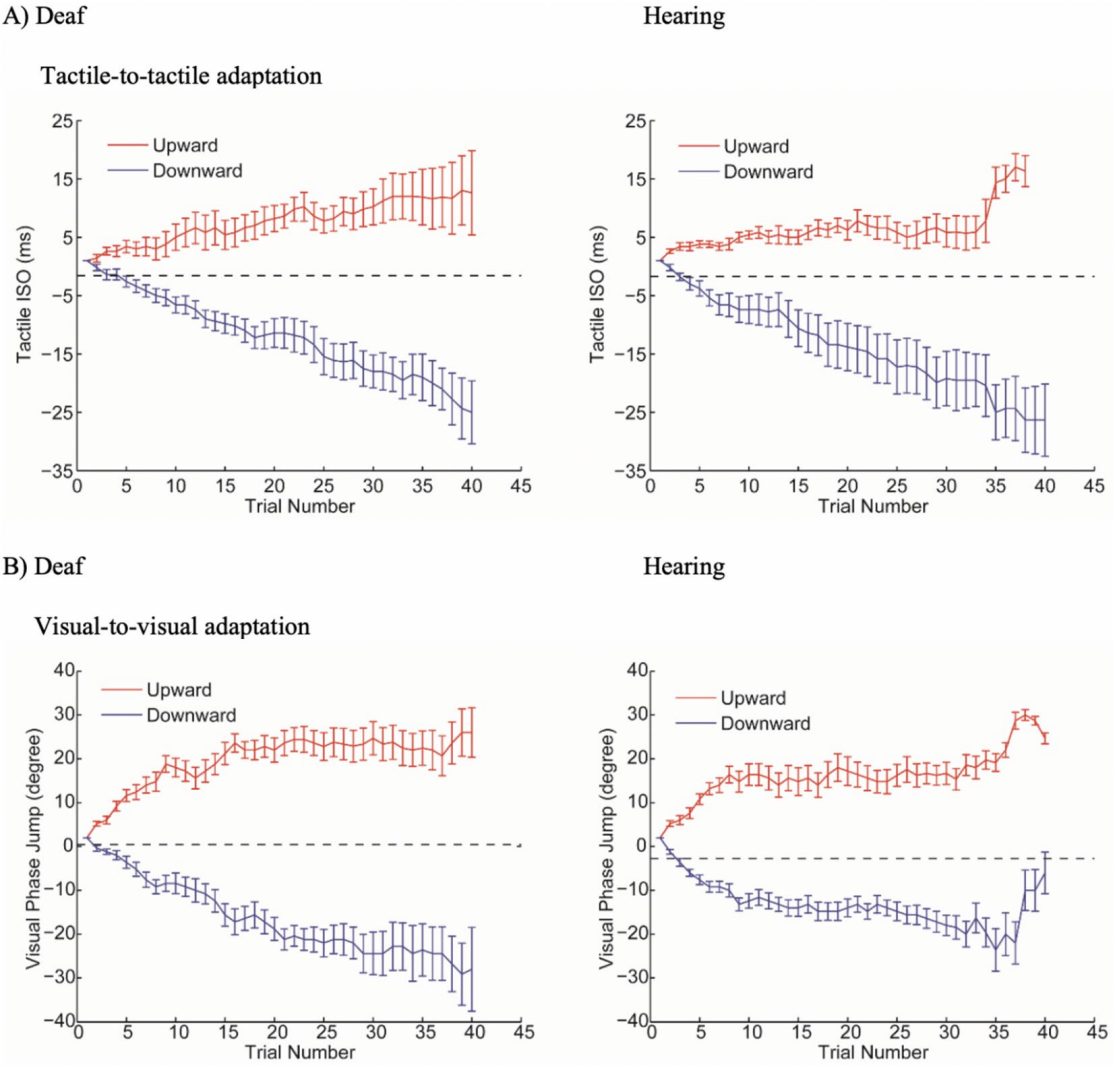
Staircase progress in Experiment 2. The staircase progress is shown separately for the deaf (left column) and hearing participants (right column) in the tactile-to-tactile (panel **A**) and visual-to-visual (panel **B**) adaptation conditions. Staircases in the upward and downward adaptation conditions are shown respectively in red and blue. The black dashed lines were the average baseline PSE without adaptation. Error bars represent one standard error.

*Tactile-to-tactile (TT) adaptation*. The ANOVA showed a significant main effect of adapting direction (*F*(1, 18) = 41.13, *P* < 0.001, partial *η*^2^ = 0.70) but no main effect of group (*F*(1, 18) = 1.51, *P* > 0.23, partial *η*^2^ = 0.08) or interaction (*F*(1, 18) = 0.03, *P* > 0.87, partial *η*^2^ = 0.002). Post-Hoc *t*-tests showed that PSE after upward adaptation was significantly higher than that after downward adaptation for both hearing (*t*(9) = 4.24, *P* = 0.002, Cohen’s *d* = 2.16) and deaf individuals (*t*(9) = 4.94, *P* < 0.001, Cohen’s *d* = 2.74) (Fig. 8A). As shown in Fig. 9A, significant divergence between PSEs after upward and downward adaptation was observed in both hearing (Sign test, *P* = 0.002) and deaf group (Sign test, *P* = 0.002).

Compared to the baseline PSE, PSE after adaptation was significantly different for both deaf (upward adaptation: *t*(1, 9) = 4.31, *P* = 0.002, Cohen’s *d* = 1.44; downward adaptation: *t*(1, 9) = 4.19, *P* < 0.003, Cohen’s *d* = 1.39) and hearing group (upward adaptation: *t*(1, 9) = 3.85, *P* < 0.004, Cohen’s *d* = 1.28; downward adaptation: *t*(1, 9) = 3.35, *P* < 0.009, Cohen’s *d* = 1.12) (Fig. 8A). There was no significant difference of baseline PSE between the two groups (*P* > 0.80).

*Visual-to-visual (VV) adaptation*. The ANOVA showed a significant main effect of adapting direction (*F*(1, 18) = 94.38, *P* < 0.001, partial *η*^2^ = 0.84) but no main effect of group (*F*(1, 18) = 0.006, *P* = 0.94, partial *η*^2^ < 0.001) or interaction (*F* (1, 18) = 2.03, *P* > 0.17, partial *η*^2^ = 0.1). Post-Hoc t-tests showed that PSE after upward adaptation was significantly higher than that after downward adaptation for both hearing (*t*(9) = 8.71, *P* < 0.001, Cohen’s *d* = 2.65) and deaf individuals (*t*(9) = 6.33, *P* < 0.001, Cohen’s *d* = 2.94). The staircases progress after upward and downward motion adaptation showed reliable divergence for both groups (Sign test, deaf: *P* < 0.003, hearing: *P* < 0.003) (Fig. 9B).

PSE after adaptation was significantly different from the baseline PSE, for both the deaf (upward adaptation: *t*(1, 9) = 4.05, *P* < 0.003, Cohen’s *d* = 1.35; downward adaptation: *t*(1, 9) = 6.49, *P* < 0.0002, Cohen’s *d* = 2.16) and hearing group (upward adaptation: *t*(1, 9) = 6.33, *P* < 0.0002, Cohen’s *d* = 2.11; downward adaptation: *t*(1, 9) = 3.30, *P* < 0.01, Cohen’s *d* = 1.10) (Fig. 8B). There was no significant difference of baseline PSE between the two groups (*P* > 0.20).

*Tactile and visual discrimination thresholds of motion-direction*. Tactile motion thresholds of the deaf (*M* = 11.09, *SD* = 4.26) and those of the hearing (*M* = 13.35, *SD* = 2.93) were not significantly different (*t*(18) = 1.38, *P* > 0.18, Cohen’s *d* = 0.61). Similarly, there was no significant difference in discrimination thresholds for visual motion between the deaf (*M* = 9.43, *SD* = 4.46) and hearing (*M* = 10.81, *SD* = 7.12) (*t*(18) = 0.52, *P* > 0.60, Cohen’s *d* = 0.23).

## Discussion

In the current study, we examined the effects of early auditory deprivation on the visuo-tactile interaction by comparing visuo-tactile cross-modal aftereffects between deaf and hearing participants. Consistent with the previous study by Konkle et al.^31^, we found bidirectional cross-modal motion aftereffects in the hearing group: aftereffects transferred from vision to touch and from touch to vision. In contrast, the deaf group showed a unidirectional cross-modal motion aftereffect: the aftereffect transferred only from touch to vision but not from vision to touch. In addition, we found no difference in unisensory motion aftereffects or in discrimination thresholds of motion-direction between the hearing and deaf group. Thus, the observed difference in cross-modal motion aftereffects between hearing and deaf participants cannot be attributed to unisensory motion aftereffects or motion direction discrimination thresholds.

The reliance on tactile over visual modality observed in deaf individuals for tactile motion perception is in line with the modality appropriateness hypothesis that sensory information is weighted according to the relative precision of the information conveyed by each sensory modality^28^. Within this framework, the sensory modality that is most relevant in that particular context will be weighed more and thus dominate behaviors^29,30^. In the current study, tactile motion perception can be considered as weighting more on temporal processing, given that the motion direction is manipulated by varying the temporal gap between two tactile rows (i.e., ISO). Without auditory input, deaf individuals seem to rely more on touch than hearing for temporal processing, and in turn, are less influenced by vision when performing tactile motion discrimination. The dominance of touch over vision in the deaf, therefore, is embodied in a task that taps on temporal features.

The context specificity is the core character of the modality appropriateness hypothesis. Within this framework, when auditory input is absent, deaf people would rely more on touch for temporal processing. Our results are consistent with previous research showing a reduced visual-to-tactile influence in visuo-tactile integration^42^ and those reported by Karns et al.^23^ showing higher susceptibility to the double-flash visual illusion induced by two touches to the face in congenitally deaf adults, suggesting sensory modality with greater temporal precision (touch) influencing the timing of a less precise modality (vision). In contrast, increased visual interference during visuo-tactile spatial interference tasks was reported in deaf individuals as compared to hearing controls^24^, suggesting sensory modality with greater spatial precision (vision) influencing the location of a less precise modality (touch). Combined, these results are suggestive of a double dissociation, whereby in deaf individuals the influence of touch is enhanced in temporal tasks and that of vision is enhanced in spatial tasks. Therefore, preferences to process a given class of stimuli via a specific sensory modality may be enhanced as a result of sensory deprivation.

It is noteworthy that the relatively increased weighting of the touch over vision was not accompanied by an enhanced tactile motion processing, as the deaf showed no better sensitivity to tactile motion direction. In addition, no difference of tactile-to-tactile aftereffects was found between the deaf and the hearing group, suggesting that tactile intra-modal motion processing did not benefit from auditory deprivation. Similarly, previous studies indicate that deaf individuals show no enhancement or even impairment in tactile temporal processing^13–17^. The results presented here, on the other hand, can be interpreted instead as relative robustness of the touch over vision—deaf individuals are less influenced by visual stimulation and thus able to filter out distracting information from the visual modality and report the actual direction of the tactile motion. This relative robustness, however, is largely driven by the particular context (i.e., the nature of the task) and seems to be independent of tactile intra-modal sensitivity.

It is reasonable to conclude that the visual-to-tactile motion aftereffect is at least reduced after hearing loss. Admittedly, although our power analysis based on the preceding study by Konkle et al.^31^ showed that a sample size of ten had an acceptable level of power due to the large effect size of visuo-tactile aftereffect, the conclusion drew from a small sample size should be taken with caution, and the reported unidirectional visuo-tactile aftereffects found in the deaf individuals shall be examined with a larger sample size in the future. Besides, further research may consider adding a visual-to-tactile synchronization phase in the practice session in the visual-to-tactile condition, as in the tactile-to-visual condition. The reason for not including the visual-to-tactile synchronization in the Konkle et al. study^31^ or the present study was that previous evidence already showed strong visual-to-tactile influence, and both studies found significant visual-to-tactile aftereffects in hearing individuals. Nevertheless, the lack of a visual-to-tactile synchronization procedure during the practice session may have potentially contributed to the lack of visual-to-tactile aftereffects in the deaf group. To further understand visuo-tactile interaction in deaf individuals, it would be helpful to investigate factors (e.g., stimulus intensity) that influence the observed unidirectional cross-modal motion aftereffect in future research.

## Supplementary Information

Supplementary Information

## Data Availability

De-identified datasets generated or analyzed during the current study are available from the corresponding authors on reasonable request.
